# Evaluations of the Antimicrobial Activities and Chemical Compositions of Body Fat from the Amphibians *Leptodactylus macrosternum* Miranda-Ribeiro (1926) and *Leptodactylus vastus* Adolf Lutz (1930) in Northeastern Brazil

**DOI:** 10.1155/2013/913671

**Published:** 2013-04-23

**Authors:** Mario Eduardo Santos Cabral, Diógenes de Queiroz Dias, Débora Lima Sales, Olga Paiva Oliveira, Diego Alves Teles, João Antonio de Araujo Filho, José Guilherme Gonçalves de Sousa, Henrique Douglas Melo Coutinho, José Galberto Martins da Costa, Marta Regina Kerntopf, Rômulo Romeu da Nóbrega Alves, Waltécio de Oliveira Almeida

**Affiliations:** ^1^Laboratory of Zoology, Regional University of Cariri-URCA, Pimenta, 63105-000 Crato, CE, Brazil; ^2^Laboratory of Microbiology and Molecular Biology, Regional University of Cariri-URCA, Pimenta, 63105-000 Crato, CE, Brazil; ^3^Laboratory of Natural Products Research, Regional University of Cariri-URCA, Pimenta, 63105-000 Crato, CE, Brazil; ^4^Laboratory of Phamacology and Medicinal Chemistry, Regional University of Cariri-URCA, Pimenta, 63105-000 Crato, CE, Brazil; ^5^Department of Biology, Paraiba State University-UEPB, 58429-500 João Pessoa, PB, Brazil

## Abstract

*Leptodactylus macrosternum* and *L. vastus* (family: Leptodactylidae) are commonly encountered in the “Caatinga” biome in northern Brazil. The body fat of *L. vastus* is used as a zootherapeutic for treating a number of human maladies. The aim of this work was to determine the chemical composition of the body fats of *L. macrosternum* and *L. vastus* and to evaluate their antimicrobial activities as well as the ecological implications of their use in traditional folk medicine. Oils were extracted from body fat located in the ventral region of *L. macrosternum* (OLM) and *L. vastus* (OLV) using hexane as a solvent. The fatty acids were identified by GC-MS. The antimicrobial activities of the oils, either alone or in combination with antibiotics and antifungal drugs, were tested on standard strains of microorganisms as well as on multiresistant strains of *Escherichia coli* and *Staphylococcus*. OLM contained 40% saturated and 60% unsaturated fatty acids, while OLV contained 58.33% saturated and 41.67% unsaturated fatty acids. Our results indicated that both OLM and OLV demonstrated relevant antimicrobial activities (with MIC 256 **μ**g/mL for both) against *Pseudomonas aeruginosa* and *Candida krusei*. However, no antimicrobial effects were observed when these oils were combined with antibiotics or antifungal drugs.

## 1. Introduction 

Brazil is culturally diverse and has an extremely wide variety of animal species that are used by humans in many different ways [1–3]. Many animals and plants are used as ingredients of folk remedies in traditional medicinal practices in northeastern Brazil [4], and ethnozoological researchers have documented their open commercialization in public markets in that region [5–7].

Many natural products have been investigated as promising sources of new drugs [8, 9] and increasing attention has been given to both vertebrate and invertebrate animals as potential sources of these medicines [10]. These possible products can represent secondary metabolites or proteins, as squalamine, magainins and others [11–13]. Hunt and Vincent [14] and Mayer and Gustafson [15] noted that many faunal resources have been tested for extractable bioactive compounds, and pharmaceutical companies have isolated significant numbers of substances derived from animals in the search for new drugs [16] and many are now used to produce essential medicines [17]. Rashid et al. [18], for example, isolated and purified a polysaccharide from the sponge *Celtodoryx girardae* that demonstrated important antiviral activity against *Herpes simplex*; Stankevicins et al. [19] evaluated the antimutagenic activity of extracts of the sponge *Arenosclera brasiliensis*; and De Barros et al. [20] isolated a type of heparin from the ascidia *Styela plicata* that demonstrated significant anesthetic properties. 

Amphibians are generally only infrequently mentioned [4] among the vertebrates used in Brazilian folk medicine, although species of the genera *Leptodactylus* (Leptodactylidae) and *Rhinella* (Bufonidae) have received a fair number of citations [5–7, 17, 21–25].

The family Leptodactylidae comprises four genera and 100 species, and the genus *Leptodactylus* comprises 89 species distributed throughout South America, principally in Brazil and the Antilles [26]. *Leptodactylus macrosternum* belongs to the *Leptodactylus ocellatus* group (Linnaeus, 1758), which is the smallest genus of the family (with only six species) although there are various taxonomic problems associated with this taxon [27]. *L. macrosternum* is widely distributed throughout South America (east of the Andes Mountains) and occurs from Venezuela to Argentina, including Brazil [28]; *it *is considered a generalist species that is well adapted to disturbed areas and its habitats vary from open, dry environments to humid tropical forests [29].

*L. vastus* is endemic to South America and is widely distributed throughout northeastern Brazil [30] where it is popularly known as “jia” or “Northeastern pepper frog.” It is a large animal that inhabits freshwater and terrestrial environments [31] and deposits its foam egg masses in freshwater sources [32]. In spite of their ample distributions in northeastern Brazil, only a few ethnozoological surveys have mentioned the medicinal use of the body fat of *L. vastus* [7, 21–23]. Ferreira et al. [23] noted that the body fat of *L. vastus* is used to treat throat inflammations, coughs, asthma, arthritis, and sore backs. 

While there are no available citations of the medicinal uses of *L. macrosternum*, its investigation in the present study is justified following the chemotaxonomic method—which takes into account phylogenetic relationships between organisms and uses this information to initiate investigations of the pharmacological properties of related taxa [33]. 

The present study reports the identification of the chemical constituents of the fixed oils of *L. macrosternum* and *L. vastus* and the evaluations of their antimicrobial activities (when administered individually or in combination with antibiotics and antifungal drugs).

## 2. Materials and Methods

### 2.1. Collecting the Amphibian Specimens

**Figure 1:**
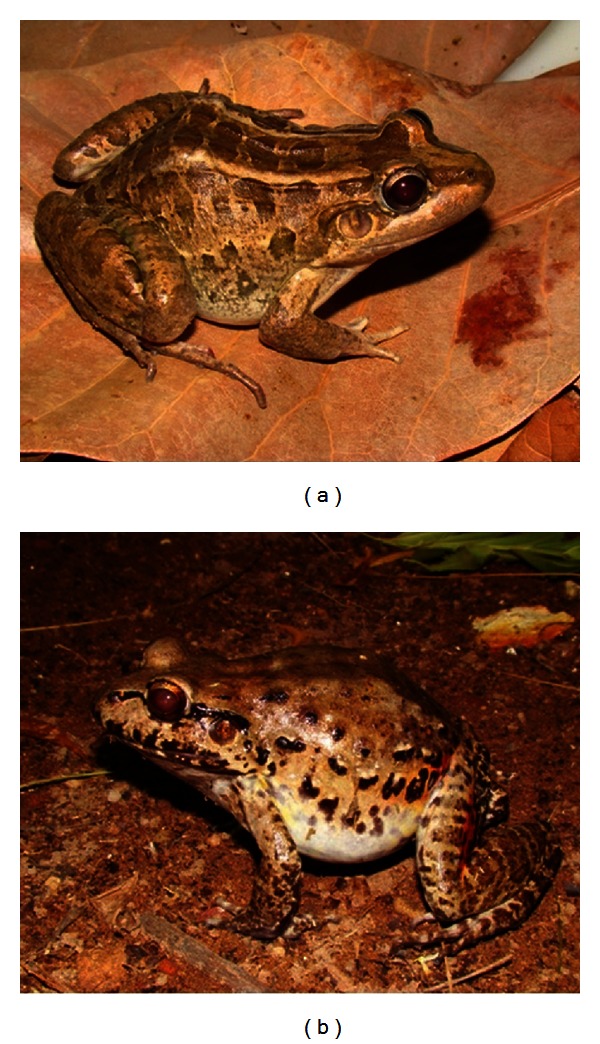
Species of frogs used in traditional medicine. (a) *Leptodactylus macrosternum* and (b) *Leptodactylus vastus* (Photos: (a), (b) Robson Waldemar Ávila).

Specimens of *L. macrosternum* and *L. vastus* (Figure 1) were collected in the Aiuaba Biological Station (06°36′–06°44′S and 40°07′–40°19′W) in the Sertão dos Inhamuns microregion, Ceará State, Brazil. The collections are made in May 2011 using active collection techniques, as described by Auricchio and Salomão [34]. The captured frogs were anesthetized with a combination of ketamine (60 mg/kg) and xylazine (6 mg/kg) [35] and subsequently sacrificed to remove their body fat. Testimonial specimens were fixed with 10% formol and subsequently deposited in the herpetological collection of the Universidade Regional do Cariri/LZ-URCA (registry numbers LZ-1325 and LZ-1309 for the species *L. macrosternum* and *L. vastus,* resp.).

### 2.2. Extraction of the Fixed Oils of *L. macrosternum* (OLM) and *L. vastus* (OLV)

The fixed oils present in body fat in the ventral regions of these frogs were extracted with hexane (60°C) for 6 h in a Soxhlet apparatus. The hexane was subsequently decanted and filtered, and the solvent removed using a rotary evaporator under reduced pressure and controlled temperature conditions (70°C ± 2°C). The quantities of fats and the oil volumes and yields are listed in Table 1.

### 2.3. Identification of the Fatty Acids

The fatty acids were identified indirectly using their corresponding methyl esters. The extracted oils (0.2 g) were saponified by refluxing for 30 min. in a solution of potassium hydroxide and methanol, following the methodology described by Hartman and Lago [36]. The pHs of the extracts were adjusted, and the free fatty acids were subsequently methylated by acid catalysis to obtain their methyl esters. 

### 2.4. Gas Liquid Chromatography (GLC) Analysis

The analysis of volatile constituents was carried out in a Hewlett-Packard GC/MS, model 5971, using the nonpolar fused silica column DB-1 (30 m × 0.25 mm i.d., 0.25 *μ*m film), eluted with helium gas at 8 mL/min with split mode. Injector and detector temperatures were set to 250°C and 200°C, respectively. The column temperature was programmed from 35°C to 180°C at 4°C/min and then from 180°C to 250°C at 10°C/min. Mass spectra were recorded from 30 to 450 *m*/*z*, with an electron beam energy of 70 eV. The individual components were identified by computer MS library searches, using retention indices as a preselection routine, and visual inspection of the mass spectra from the literature for confirmation [37], as well as by visually comparing standard fragmentation to that reported in the literature [38, 39].

### 2.5. Microorganisms

Experiments were undertaken using clinical isolates of *Escherichia coli* (EC27) resistant to neomycin and gentamicin (low levels), tobramycin, amikacin, and kanamycin, *Staphylococcus aureus* 358 (SA358) resistant to various aminoglycosides, and *Pseudomonas aeruginosa* (PA22). *E. coli* ATCC 10536, *S. aureus* ATCC 25923, *P. aeruginosa* ATCC 15442, and *Klebsiella pneumoniae* ATCC 4362 were used as positive controls. To evaluate antifungal activity, isolates of *Candida albicans* ICB 12 and *C. krusei* ATCC lineage 6258 were used. All of the lineages were maintained in heart infusion agar slants (HIA, Difco). The cells were cultivated during the night before the trials at 37°C in a Brain Heart Infusion medium (BHI, Difco).

### 2.6. Drugs

The antibiotics gentamicin, amikacin, and neomycin were obtained from Sigma Chemical Corp., St. Louis, MO, USA. The antifungal drugs used were amphotericin B (Sigma Co., St. Louis, USA), Mebendazol (Lasa Pharmaceutical Industries LTDA, Brazil), nystatin (Laboratório Teuto Brasileiro S/A, Brazil), and metronidazole (Prati, Donaduzzi & Cia LTDA, Brazil). All of these compounds were dissolved in sterile water before use.

### 2.7. Determination of the Minimum Inhibitory Concentrations (MICs) and Modulatory Activities

**Figure 2:**
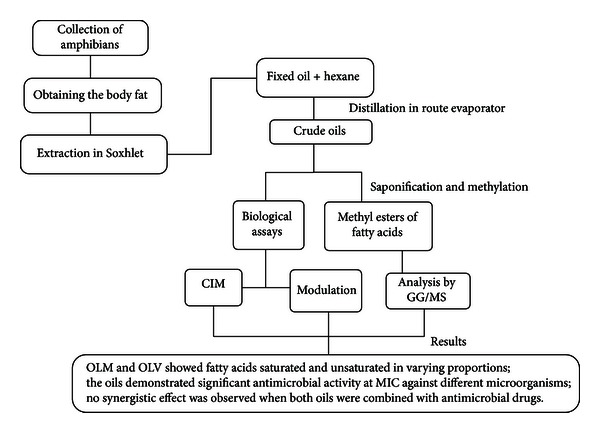
General diagram of the method of extraction of oils from *L. macrosternum* and *L. vastus* and results in biological assays.

The MIC of the oils of *L. macrosternum* and *L. vastus*, antibiotics, and antifungal agents were determined in BHI by microdilution using suspensions of 10^5^ CFU/mL, with antibiotic concentrations varying from 2500 to 2.44 *μ*g/mL and antifungal drug concentrations varying from 512 to 8 *μ*g/mL (double serial dilutions) [40]. The MIC was defined as the lowest concentration of a test compound that could inhibit bacterial growth. To evaluate the effects of the oils as modulators of antibiotic and antifungal activities, the MICs of the antibiotics at subinhibitory concentrations were determined in the presence of the oils extracted from *L. macrosternum* and *L. vastus* (32 and 64 *μ*g/mL, resp.), as were the MICs of the antifungal agents (64 and 32 *μ*g/mL, resp.); the plates were incubated for 24 hours at 37°C (Figure 2).

## 3. Results

The methyl esters of the fatty acids from the fixed oils of the two anuran species examined were analyzed using GC/MS. Ten chemical constituents were identified in the oil extracted from *L. macrosternum* (representing fully 98.98% of the constituents) and 12 constituents (92.36%) in the oil extracted from *L. vastus* (Table 2).

The OLM contained 40% saturated fatty acids and 60% unsaturated fatty acids; the principal constituents were (8E, 11E, 14E)-docosatrienoic acid methyl ester (33.55%), palmitoleic acid methyl ester (31.52%), linoleic acid methyl ester (16.46%), and stearic acid methyl ester (7.05%). The OLV contained 58.33% saturated fatty acids and 41.67% unsaturated fatty acids; the principal constituents were elaidic acid methyl ester (27.87%), palmitoleic acid methyl ester (20.01%), linoleic acid methyl (17.02), stearic acid methyl ester (10.01%), and palmitoleic acid methyl ester (9.23%). 

The *in vitro* antimicrobial tests indicated that both oils had inhibitory effects on at least some of the microorganisms tested. The OLM demonstrated antibacterial activity against *P. aeruginosa* ATCC 15442 (with an MIC of 256 *μ*g/mL). This MIC was similar to that observed when OLV was administered to *C. krusei* ATCC 6258 (Table 3). However, neither of the oils demonstrated antimicrobial activity at clinically relevant concentrations against *E. coli* ATCC 10532, *S. aureus* ATCC 25923, *K. pneumoniae* ATCC 4362, or *C. albicans* ICB 12 (MIC ≥ 1024 *μ*g/mL). These results indicated that both oils were effective in inhibiting opportunist microorganisms and that they could be used as alternative sources of treatments for illnesses such as sore throats caused by bacterial infections. 

Tests of the abilities of these oils to modify antibiotic activities revealed the absence of any inhibitory activity against any of the bacterial lineages when OLM was combined with the aminoglycosides tested. Some antagonistic effects were observed against *S. aureus* 358 and *P. aeruginosa* 22, however, when OLV was associated with neomycin (Table 4). 

Neither oil demonstrated clinically relevant activity in modulating the effects of antifungal drugs, with MIC values ≥1024 *μ*g/mL. These results indicated a lack of efficiency of *L. macrosternum* and *L. vastus* fat associated with antibiotics or antifungal agents in treating illnesses caused by opportunist bacteria or fungi.

## 4. Discussion

The presence of large quantities of unsaturated fatty acids in both OLM and OLV was quite unexpected as these essential fatty acids are not synthesized by animals. The linoleic acid in the oils extracted from both species may have been acquired through their diets. The presence of a number of these fatty acids in both of the oils assayed here was similar to the results reported by Lopes et al. [41] for oils extracted from the adipose tissue of the amphibian *Rana catesbeiana* SHAW (including stearic, linoleic, myristic, palmitic, and palmitoleic acids). The myristic acid content (1.8%) reported by these same authors was essentially equal to that found in both of the oils analyzed in the present work, although the level of linoleic acid (25%) from *R. catesbeiana* was significantly greater than that found in both OLM (16.46%) and OLV (17.02%).

The fatty acids lauric, palmitic, linoleic, linolenic, stearic, myristic, and caprylic are known to have antibacterial and antifungal properties [42, 43]. Silva et al. [44] demonstrated the efficiency of oil extracted from *Rana catesbeiana* in inhibiting different pathological organisms, and these authors reported that this natural product was very active against all of the microorganisms tested (*S. aureus*, *E. coli*, *P. aeruginosa*, *C. albicans*, *C. tropicalis*, and *C. guilliermondii*)—indicating that it is a promising antimicrobial agent, especially in light of the fact that it was relatively easy to obtain relatively large quantities of this oil. Zheng et al. [45] suggested that the antibacterial activities of fatty acids (principally unsaturated varieties) may be due to their effects on bacterial synthesis of endogenous fatty acids. 

Granowitz and Brown [46] reported antagonistic effects from the combined use of antibiotics, which they attributed to mutual chelation. Similar antagonistic effects may be diminishing the activities of the aminoglycosides when combined with OLV in the present study.

Relatively few studies have been undertaken to examine the capacities of zootherapeutics to modify the actions of antibiotics or antifungal agents. Ferreira et al. [47] evaluated the modulatory activity of fat derived from *Tupinambis merianae* on aminoglycosides and determined that the body fat of this lizard did not increase their efficiency against the bacterial strains tested. Combinations of amikacin and neomycin with OTM did not increase their effectiveness against *E. coli* 27 or *S. aureus* 358, but this same oil demonstrated antagonistic effects with kanamycin and gentamicin against these same bacteria.

The use of natural products in association with industrialized medicines has been well documented in the scientific literature. Calvet-Mir et al. [48] reported the use of traditional folk medicines in association with western pharmaceuticals to treat diarrhea, vomiting, and stomachaches among individuals of the Tsimane ethnic group in the provinces of Ballivian and Yacuma in Bolivia. Vandebroek et al. [49] reported that rural communities in Quechua, Bolivia, used combinations of natural products and industrialized medicines to treat illnesses of the respiratory and digestive tracts—demonstrating that at least some communities utilize natural and industrialized medicines simultaneously. 

The present report is the first investigation of the use of natural products derived from *L. macrosternum* and *L. vastus* to modulate the effects of antibiotic and antifungal compounds on standard and multiresistance microorganisms. 

Hunt and Vincent [14] warned that bioprospecting for pharmaceuticals could result in the overexploitation of regional biodiversity, with strong direct and negative effects on these living resources. As such, pharmacological testing of products derived from animal species (whether threatened or not by extinction) will require proactive measures to guarantee their rational and sustainable use and the perpetuation of the species [50, 51]. 

Our results demonstrated that OLM and OLV are efficient antimicrobial agents—and it will therefore be necessary to guarantee the rational harvesting of *L. macrosternum* and *L. vastus* in order to avoid exerting excessive pressure on their natural populations. The indiscriminate use of native species for medicinal purposes has been cited as one of probable causes of the population declines in a number of plant and animal species [1, 17, 52], even though there is no actual data available that could confirm the efficiency or safety of the use of products derived from those organisms.

## 5. Conclusions

The oils derived from the body fat of *L. macrosternum* and *L. vastus* demonstrated relevant antimicrobial activities against *P. aeruginosa* and *C. krusei*, respectively; they did not, however, demonstrate clinically satisfactory inhibitory effects when combined with antibiotics or antifungal drugs. The use and sale of products derived from these amphibians could result in excessive harvesting pressure on natural wild populations. As such, we recommend (i) the elaboration of proactive management plans for the rational and sustainable use of these species and (ii) undertaking additional studies on the usefulness of the body fat of *L. macrosternum* and *L. vastus* in treating other human infirmities.

## Figures and Tables

**Table 1:** Data relating to obtaining fixed oils from the species studied.

Species	(1)	(2)	(3)
*L. macrosternum* (OLM)	11.56	4.10	36.55
*L. vastus* (OLV)	60.47	11.03	18.24

(1) fat fresh weight (g); (2) Volume (mL) of the extracted oils; (3) Oil yields (%); OLM: fixed oil of *L. macrosternum*; OLV: fixed oil of *L. vastus*.

**Table 2:** Methyl esters identified in the fixed oils in the body fat of *Leptodactylus macrosternum* (oil I) and *Leptodactylus vastus* (oil II), with their respective percentages.

Components	Oil I		Oil II	
	RI^a^	(%)	RI^a^	(%)
Myristic acid methyl ester	1680	1.16	1680	1.42
Pentadecanoic acid methyl ester	1779	0.53	1779	1.74
Palmitoleic acid methyl ester	1878	31.52	1878	9.23
Palmitic acid methyl ester	1978	0.75	1886	20.01
Oleic acid methyl ester	2085	0.49	—	—
Linoleic acid methyl ester	2077	16.46	2093	17.02
Isoheptadecanoic acid methyl ester	—	—	1914	0.69
Heptadecanoic acid methyl ester	—	—	1978	0.66
Eicosanoic acid methyl ester	—	—	1986	0.93
Elaidic acid methyl ester	—	—	2085	27.87
Stearic acid methyl ester	2241	7.05	2241	10.01
5,8,11,14-eicosatetraenoic acid methyl ester	2499	0.65	2308	2.51
4,7,10,13,16,19-docosahexaenoic acid methyl ester	—	—	2523	0.27
(8E,11E,14E)-docosatrienoic acid methyl ester	2093	33.55	—	—
(E,E,Z)-1,3,12-nonadecadienoic acid methyl ester-5,14-diol	2308	6.82	—	—

Total identified		98.98		92.36
Saturated esters		40		58.33
Unsaturated esters		60		41.67

**Table 3:** MIC values (*μ*g/mL) of the fixed oils of *Leptodactylus macrosternum* and *Leptodactylus vastus* applied to standard and multiresistant microorganisms.

Microorganisms	MIC (*μ*g/mL)	
	*L. macrosternum *	*L. vastus *
	OLM	OLV
*E. coli* ATCC 10532	≥1024	≥1024
*S. aureus* ATCC 25923	≥1024	≥1024
*K. pneumonia* ATCC 4362	≥1024	≥1024
*P. aeruginosa* ATCC 15442	256	512
*C. albicans* ICB 12	≥1024	≥1024
*C. krusei* ATCC 6258	512	256

MIC: minimum inhibitory concentration; OLM: oil from *L. macrosternum*; OLV: oil from *L. vastus.*

**Table 4:** Minimum inhibitory concentrations (*μ*g/mL) of the aminoglycosides alone and in association with the fixed oils of *L. macrosternum* and *L. vastus*.

Antibiotics	*L. macrosternum *					
	MIC SA 358	OLM (32 *μ*g/mL) + antibiotic	MIC EC 27	OLM (32 *μ*g/mL) + antibiotic	MIC PA 22	OLM (32 *μ*g/mL) + antibiotic
Amikacin	78.1	78.1	9.8	9.8	156.2	156.2
Neomycin	78.1	78.1	4.9	4.9	156.2	156.2
Gentamicin	9.8	9.8	2.4	2.4	39.1	39.1

Antibiotics	*L. vastus *					
	MIC SA 358	OLV (64 *μ*g/mL) + antibiotic	MIC EC 27	OLV (64 *μ*g/mL) + antibiotic	MIC PA 22	OLV (64 *μ*g/mL) + antibiotic

Amikacin	39.1	39.1	9.8	9.8	156.2	156.2
Neomycin	9.8	78.1	2.4	2.4	39.1	156.2
Gentamicin	4.9	4.9	2.4	2.4	39.1	39.1
